# Isolation, characterization and application of theophylline-degrading *Aspergillus* fungi

**DOI:** 10.1186/s12934-020-01333-0

**Published:** 2020-03-19

**Authors:** Binxing Zhou, Cunqiang Ma, Tao Xia, Xiaohong Li, Chengqin Zheng, Tingting Wu, Xiaohui Liu

**Affiliations:** 1 Long Run Pu-erh Tea College, Yunnan Agricultural University, Kunming, 650201 Yunnan China; 2 Kunming Dapu Tea Industry Co., LTD, Kunming, 650224 Yunnan China; 3 State Key Laboratory of Tea Plant Biology and Utilization, Anhui Agricultural University, Hefei, 230036 Anhui China

**Keywords:** *Aspergillus*, Theophylline, 3-Methylxanthine, Bioconversion, Tea, Pathway

## Abstract

**Background:**

Caffeine, theobromine and theophylline are main purine alkaloid in tea. Theophylline is the downstream metabolite and it remains at a very low level in *Camellia sinensis*. In our previous study, *Aspergillus sydowii* could convert caffeine into theophylline in solid-state fermentation of pu-erh tea through *N*-demethylation. In this study, tea-derived fungi caused theophylline degradation in the solid-state fermentation. The purpose of this study is identify and isolate theophylline-degrading fungi and investigate their application in production of methylxanthines with theophylline as feedstock through microbial conversion.

**Results:**

Seven tea-derived fungi were collected and identified by ITS, β-tubulin and calmodulin gene sequences, *Aspergillus ustus*, *Aspergillus tamarii*, *Aspergillus niger* and *A. sydowii* associated with solid-state fermentation of pu-erh tea have shown ability to degrade theophylline in liquid culture. Particularly, *A. ustus* and *A. tamarii* could degrade theophylline highly significantly (p < 0.01). 1,3-dimethyluric acid, 3-methylxanthine, 3-methyluric acid, xanthine and uric acid were detected consecutively by HPLC in *A. ustus* and *A. tamarii*, respectively. The data from absolute quantification analysis suggested that 3-methylxanthine and xanthine were the main degraded metabolites in *A. ustus* and *A. tamarii*, respectively. 129.48 ± 5.81 mg/L of 3-methylxanthine and 159.11 ± 10.8 mg/L of xanthine were produced by *A. ustus* and *A. tamarii* in 300 mg/L of theophylline liquid medium, respectively.

**Conclusions:**

For the first time, we confirmed that isolated *A. ustus*, *A. tamarii* degrade theophylline through *N*-demethylation and oxidation. We were able to biologically produce 3-methylxanthine and xanthine efficiently from theophylline through a new microbial synthesis platform with *A. ustus* and *A. tamarii* as appropriate starter strains.

## Background

Caffeine (1, 3, 7-trimethylxanthine) is the most abundant methylxanthine alkaloids in tea, and also one of the major tea flavor content causing bitterness [1]. Caffeine was extensively established to be the final mathylxanthine product biosynthesized through three steps of the methylation of xanthosine in the root of tea tree [2]. Until now, caffeine catabolism has been relatively understood and established in plants, mainly in tea (*Camellia sinensis*) and caffeine (*Coffea arabica*) [3]. The major catabolic pathway of caffeine is as follows: caffeine → theobromine/theophylline → 3-methylxanthine → xanthine → uric acid → allantoin → allantoic acid → CO_2_ + NH_3_ [4, 5]. The other alternative catabolic pathways have been reported recently in *Camellia* plants [6, 7]. Theophylline (1,3-dimethylxanthine) is a transient metabolite through the demethylation of caffeine at the position N-7 and stays a very low level due to the slow metabolism in tea leaves [8, 9].

Although caffeine level remains stable in the processing of general teas (green tea, black tea, oolong tea and white tea) [10, 11], several microorganisms selected from the soil of tea and coffee plantations could degrade caffeine, which included *Pseudomonas* sp. [12, 13], *Pseudomonas putida* [14, 15], *Serratia marcescens*, *Fusarium solani* [16, 17], *Stemphyllium* sp., *Aspergillus tamarii*, and *Penicillium commune* [17, 18]. Two possible mechanisms of caffeine catabolism in microorganisms are *N*-demethylation and oxidation [19]. Theophylline is the major metabolite formed in fungi through the *N*-demethylation of caffeine, which present marked differences from bacteria that theobromine (3,7-dimethylxanthine) or paraxanthine (1,7-dimethylxanthine) are major metabolites in caffeine catabolism [13, 19]. Moreover, *P. putida* and *Pseudomonas* sp. were established to not only use caffeine, theobromine, paraxanthine (1,7-dimethylxanthine) and 7-methylxanthine, but also degrade theophylline and 3-methylxanthine [14, 20] (*P. putida* and *Pseudomonas* sp. were established to use caffeine, theobromine, paraxanthine (1,7-dimethylxanthine) and 7-methylxanthine, and they also degrade theophylline and 3-methylxanthine). In addition, *Aspergillus niger*, *Talaromyces marneffei* and *Talaromyces verruculosus* isolated from cocoa pod husks were established to degrade theobromine and produce methylxanthine [21]. However, only bacterial strain *P. putida* CBB5 was confirmed to degrade theophylline via *N*-demethylation [14, 22]. Until now, the theophylline-degrading fungi and correlative metabolites were not completely definite.

Pu-erh tea is one of the most representative dark tea and natural microorganisms involved in solid-state fermentation (SSF) play an important role in tea processing [23, 24]. Microorganisms including bacteria and fungi have profound impact on substance metabolisms and correlation with quality formation of pu-erh tea [25–27]. *A. niger*, *Aspergillus tubingensis*, *Aspergillus fumigatus*, *Aspergillus acidus*, *Aspergillus awamori*, *A. tamarii*, *Blastobotrys adeninivorans*, *Candida tropicalis*, *Fusarium graminearum*, *Pichia farinosa*, *Rasamsonia byssochlamydoides*, *Rasamsonia emersonii*, *Rasamsonia cylindrospora*, *Rhizomucor pusillus*, *Rhizomucor tauricus* and *Thermomyces lanuginosus* have been detected in pu-erh tea [28–31]. Theophylline has several applications in therapeutics, especially as anti-asthmatic, anticancer, anti-cellulite and combinatorial drug [32–34]. Caffeine content fluctuates during the SSF, which has associated with the fungi appearing in SSF [35–39]. We found that theophylline content was increased significantly (p < 0.05) and *Aspergillus sydowii* caused caffeine degradation in SSF [40]. After further research, *A. sydowii* had a significant (p < 0.05) impact on caffeine metabolism and potential value in theophylline production through aerobic fermentation [41, 42].

In this study, we found that theophylline content had a highly significant (p < 0.01) decrease during the later period of SSF after a highly significant (p < 0.01) increase. Therefore, apart from an isolated caffeine-degrading fungus identified as *A. sydowii* causing the production of theophylline in SSF, theophylline-degrading fungi also could be found in SSF. In this paper, two theophylline-degrading fungi were isolated from the SSF and identified as *A. ustus* and *A. tamari* based on colonial morphology and ITS, β-tubulin and calmodulin gene sequences, respectively. Theophylline degradation metabolites and pathways were analyzed in fungi by high-performance liquid chromatography (HPLC). The application in production of methylxanthines was investigated by using *A. ustus* and *A. tamarii*, respectively.

## Methods

### Materials and reagents

Sun-dried green tea leaves (*C. sinensis* var. *assamica*) with moisture content 6.25% by weight were obtained from Yunnan Province, China. Caffeine, theophylline, 3-methylxanthine, 1-methylxanthine, xanthine, 1,3-dimethyluric acid, 1-methyluric acid, 3-methyluric acid and uric acid were purchased from USA Sigma Company. SP fungal DNA kit was purchased from USA Omega Company. DNA marker, polymerase chain reaction (PCR) spread reagent, primers [43, 44]: ITS1 (5′-TCCGTAGGTGAACCTGCGG-3′) and ITS4 (5′-TCCTCCGCTTATTGATAGC-3′); Bt2a (5′-GGTAACCAAATCGGTGCTGCTTTC-3′) and Bt2b (5′-ACCCTCAGTGTAGTGACCCTTGGC-3′); and CF1L (5′-GCTGACTCGTTGACCGAAGAG-3′) and CF4 (5′-ATTTTTGCATCATGAGCTGAAC-3′) were purchased from Japan TaKaRa Company. Other reagents were ether analytical grade or chromatographic grade.

### Pu-erh tea solid-state fermentation and determination of caffeine and theophylline

The SSF of pu-erh tea was based on the natural microbiota existing on tea leaves and fermentation environment. A 2 kg sample of sun-dried green tea leaves was mixed with 885 mL tap water to achieve given moister content of 35% (w/w) [30]. During the fermentation, tea leaves were mixed to ensure homogeneity and tap water was added to keep the appropriate moisture content at 25–35%. The whole fermentation continued for about 35 days and samples were collected every 5 days. The colony forming units (CFU) were calculated by per gram of the dry weight after 2 days of cultivation at 30 °C. Caffeine and theophylline contents were determined by HPLC described by Zhou et al. [40, 41] using an Agilent 1200 series system and an Agilent C_18_ Chromatogram column (250 mm × 4.6 mm, 5 μm). Samples collected on days 20 and 25 were stored at 4 °C and selected for further isolation and identification of theophylline-degrading fungi.

### Fungal identification of isolates

Fungal strains were isolated using potato dextrose agar (PDA) medium and they were counted by dilution-plating method [29]. Microscopic and morphological examinations of colonies were carried out according to mycological guide, and morphological features of their colonies are recorded in Table 1.

**Table 1:** Colony characteristics of theophylline-degrading fungi

Isolate	Shape	Surface	Color	Exudates	References
PT-1	Circular	Rough	Black	None	[40]
PT-2	Circular	Rough	Olive green	Red-coloured	[40]
PT-3	Circular	Rough	Dark yellow colonies with white edges	Yellow sclerotium	[41]
PT-4	Irregular	Rough	Light yellow	Yellow sclerotium	[41]
PT-5	Circular	Rough	Greyish-green centre with yellow patches	Red pigment	[41]
PT-6	Circular	Rough	Iron gray bulge with milk white edges	None	Additional file 1: Figure S1
PT-7	Irregular	Rough	Hazel green with gray back	None	Additional file 1: Figure S2

Fresh cells were obtained by centrifugation at 1700*g* for 5 min after cultivation in 20 mL of Czapek Dox medium at 30 °C for 2 days on an incubator shaker (250 rpm) and freeze-dried at − 80 °C [40, 41]. DNA was extracted by using SP fungal DNA kit. The fungal primers ITS1 and ITS4, Bt2a and Bt2b, and CF1L and CF4 were used in PCR to amplify ITS, β-tubulin and calmodulin regions, respectively [43]. The final volume of 50 μL, 1.0 μL of containing template DNA, 5 μL of 10 × buffer, 5 μL of dNTPs (2.5 mM), 0.5 μL of Taq polymerase, 1.0 μL (10 *μ*M) of each primer, and 36.5 μL of sterile distilled water were used to implement amplifications [40, 41]. The PCR reaction procedure was as follows. Pre-degeneration at 95 °C for 5 min, degeneration at 94 °C for 1 min, annealing at 54 °C for 1 min, extension at 72 °C for 1.5 min, with 35 cycles, extension at 72 °C for 10 min [44]. It was stored at 10 °C in the end of the reaction process.

The PCR was produced in an ABI3730 automatic DNA sequencer (Applied Biosystems, USA) [40]. The received sequences were sent to Genbank of NCBI to seek similar sequences of type strain by using Blastn [44]. Multiple sequence alignment was carried out by using Clustal X for Windows. The evolution distance was calculated through a Kimura2-parameter of the MEGA 4.0 Soft.

### Evaluation of growth of isolates in agar mediums

The isolate strains were transferred into PDA medium and incubated aerobically at 30 °C for 72 h on an incubator shaker (250 rpm), respectively. The spore suspension was adjusted to 1.0 × 10^7^ CFU/mL for inoculation after eluting by using sterile saline solution. Qualitative screenings were carried out in Petri dishes containing a solid culture medium contained 20 g/L agar, 4.0 g/L NaNO_3_, 1.3 g/L KH_2_PO_4_, 0.19 g/L Na_2_HPO_4_·7H_2_O, 0.26 g/L CaCl_2_·2H_2_O, 0.19 g/L MgSO_4_ and 20 g/L dextrose as carbon source (control culture) or a selection medium with theophylline instead of dextrose in three different concentrations: 600, 1200 and 1800 mg/L per plate, respectively [40]. Fungal spore suspensions were transferred to the surfaces of the agar plates with an inoculating loop (10 μL). Isolates were incubated at 30 °C for 5 days. Compared with the control culture, theophylline utilization capacity of isolates was estimated by the size of the colony grown on the plates (Table 3).

### Assessment of theophylline-degrading fungi in different theophylline liquid mediums

Theophylline liquid medium (TLM) was prepared using 4.0 g/L NaNO_3_, 1.3 g/L KH_2_PO_4_, 0.19 g/L Na_2_HPO_4_·7H_2_O, 0.26 g/L CaCl_2_·2H_2_O, 0.15 g/L MgSO_4_, 2.0 g/L sucrose and 300 mg/L theophylline in distilled water [45]. To investigate the influence of carbon and nitrogen source on theophylline degradation, the modifications used either 5 g/L sucrose or 10 g/L dextrose as carbon source in TLM with sucrose as carbon source (TLM-S) or TLM with dextrose as carbon source (TLM-D), and 1.01 g/L ammonium sulphate as nitrogen source in TLM with ammonium sulphate as nitrogen source (TLM-N), and 5 g/L sucrose and 1.01 g/L ammonium sulphate in TLM with sucrose and ammonium sulphate as carbon and nitrogen sources (TLM-SN), respectively. The spore suspension was adjusted to 1.0 × 10^7^ CFU/mL and all given TLM was adjusted for pH 6.0 by phosphate buffer before inoculation. For each isolate, control and experimental mediums (25 mL each) were inoculated with spore suspension with 4% inoculum size (v/v) that 1 mL spore suspension was inoculated into each medium, and biocidal treatment was defined as the control. Theophylline concentration was determined and mycelium was collected after cultivation at 30 °C for 5 days on an incubator shaker (130 rpm), respectively. The collected mycelium was filtered in a Buchner funnel, and rinsed in 20 mL of water: ethyl acetate (1:1) [46]. The mycelial mass was determined as fungal dry mass after drying at 35 °C for 24 h and results were summarized in Additional file 2: Table S1 [46]. Theophylline concentration was determined by HPLC [21].

### Analysis of theophylline degradation metabolites by selected isolates

Through comparison, additional sucrose could promote theophylline degradation in liquid culture. Therefore, TLM-S was selected as the optimal medium to analyze theophylline degradation by selected isolates. A series of TLM-S mediums with different initial theophylline concentrations (100, 200 and 300 mg/L, respectively) were set up each day of incubation and a 7-day period cultivation of each selected isolates were carried out on an incubator shaker (130 rpm, 30 °C). At intervals of up to 24 h for 7 days, an aliquot of each culture was filtered through a 0.45 μm syringe filter, and theophylline concentration and related metabolites were determined by HPLC according to the method from Mensah et al. [21].

Standard calibration curves were prepared from solutions of theophylline, 3-methylxanthine, 1-methylxanthine, xanthine, 1,3-dimethyluric acid, 1-methyluric acid, 3-methyluric acid and uric acid. 2 mL aliquots from each collected culture were filtered and analyzed by HPLC for theophylline, 3-methylxanthine, 1-methylxanthine, xanthine, 1,3-dimethyluric acid, 1-methyluric acid, 3-methyluric acid and uric acid. Internal standard method was used to aid in identification of metabolites. The concentrations of main degradation products (3-methylxanthine and xanthine) were analyzed in inoculated culture medium by selected isolates.

### Influence of selected isolates on 3-methylxanthine and xanthine

Analysis of theophylline degradation metabolites showed that *N*-demethylation was the main theophylline degradation pathway in fungi, and 3-methylxanthine and xanthine were main demethylated products. To explore the effects of selected isolates on 3-methylxanthine and xanthine, 3-methylxanthine and xanthine liquid mediums were prepared as above described with 5 g/L sucrose as carbon source and 100 mg/L 3-methylxanthine or 100 mg/L xanthine, respectively. Each isolate was inoculated with 4% inoculum size (v/v) and biocidal treatment was defined as the control. 3-Methylxanthine and xanthine concentrations were determined by HPLC after cultivation at 30 °C for 5 days on an incubator shaker (130 rpm), respectively.

### Statistical analysis

Three biological replications were carried out to ensure validity and repeatability. All data are presented as mean value ± standard deviation (SD). One-way analysis of variance (one-way ANOVA) was carried out by Duncan’s multiple-range test using SPSS 20.0 for window to determine whether the significant difference at p < 0.05 level or the highly significant difference in p < 0.01 level exist.

## Results

### Theophylline degradation exists in solid-state fermentation of pu-erh tea

**Fig. 1:**
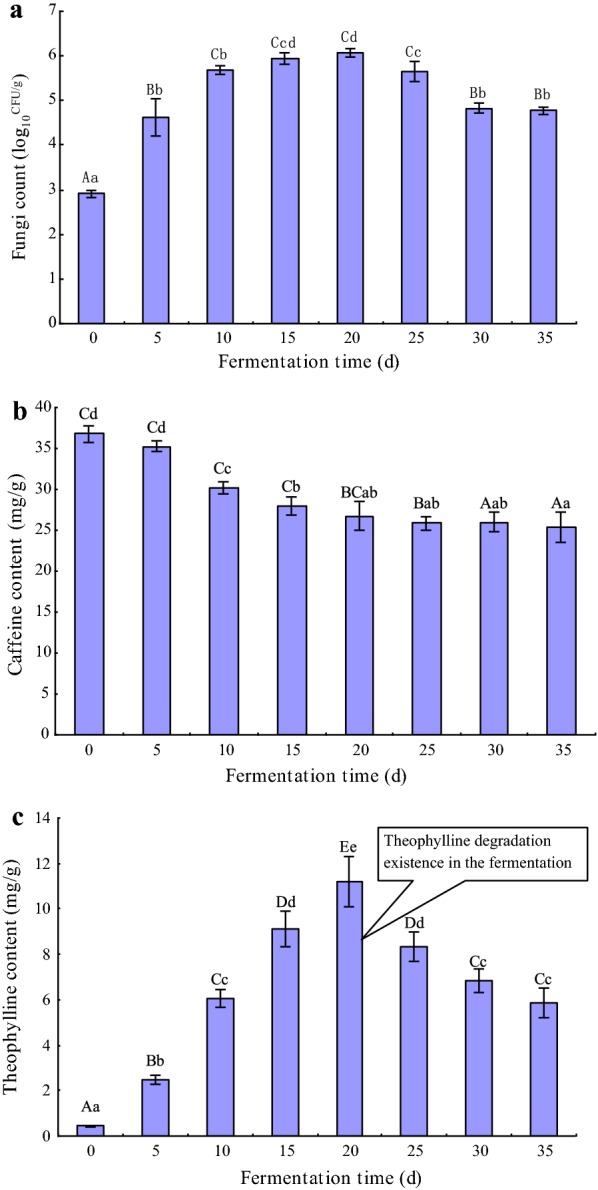
Changes of fungi count (**a**), caffeine content (**b**) and theophylline content (**c**) during the solid-state fermentation of pu-erh tea. All data were present by mean value ± SD of three replications. The lowercase letters indicated a significant difference at p < 0.05 levels and the uppercase letters indicated a highly significant difference at p < 0.01 levels using one-way ANOVA of SPSS 20.0. The different letters show significant differences

Fungi count, caffeine and theophylline contents were determined in natural SSF of pu-erh tea, and results are presented in Fig. 1. Fungi count (Fig. 1a) dramatically increased from day 0 to 10 and then increased slowly before day 20. After day 20, fungi count maintained a high level overt 1.0 × 10^5^ CFU/g. Because of the metabolic activity of fungi, caffeine content (Fig. 1b) was decreased highly significantly (p < 0.01) from 36.85 ± 1.02 to 25.46 ± 1.85 mg/g during fermentation. Theophylline content (Fig. 1c) was increased highly significantly (p < 0.01) before day 20, which confirmed that caffeine-degrading fungi leaded to caffeine degradation and theophylline production. However, after day 20, theophylline content had a highly significant (p < 0.01) decrease from 11.18 ± 1.10 to 5.89 ± 0.65 mg/g, showing that theophylline degradation appeared in SSF except for caffeine degradation. Therefore, in consideration of fungal community, there are theophylline-degrading fungi in the SSF, which could be *A. sydowii* or other fungi.

### Isolation and identification of theophylline-degrading fungi

Based on colony morphology, eleven filamentous fungi were initially selected and isolated from the SSF of pu-erh tea. Among them, seven fungi were superior in number and coded orderly with numbers PT-1 to PT-7. Distinctive morphological features of the seven isolates were observed after cultivation at 30 °C for 5 days and documented in Table 1.

The sequences obtained from the pure isolate in this study were deposited in GenBank under the accession number from MT065763 to MT065769 and from MT084116 to MT064123. Based on the DNA sequences in Table 2 and Additional file 1: Figures S3, S4, seven dominating isolates were belonged to 6 *Aspergillus* spp. and 1 *Penicillium* sp., respectively. Through neighbor-joining analysis in the phylogram for *Aspergillus* species (Additional file 1: Figures S5a, b), strain PT-6 was clustered with *A. ustus* and showed a 100% of identity to the tested *A. ustus* NRRL275; additionally, strain PT-7 was closely related to *A. tamarii* NRRL20818 with 99.9% of identity. In general, those seven candidate isolates were identified as *A. niger*, *A. sydowii*, *Aspergillus pallidofulvus*, *Aspergillus sesamicola*, *Penicillium manginii*, *A. ustus and A. tamarii* based on their morphological features and amplified sequences, respectively.

**Table 2:** Identification of theophylline-degrading fungi by sequence determination

Isolate	Primers	Fragments (bp)	Accession number^a^	Species	Strain number	Identity (%)
PT-1	ITS1/ITS4	546	MT065763	*Aspergillus niger*	NCBT 110A	99.8
PT-2	ITS1/ITS4	516	MT065764	*Aspergillus sydowii*	NRRL 250	99.8
PT-3	ITS1/ITS4	541	MT065765	*Aspergillus pallidofulvus*	NRRL 4789	99.9
	Bt2a/Bt2b	516	MT084116			
	CF1L/CF4	765	MT084120			
PT-4	ITS1/ITS4	532	MT065766	*Aspergillus sesamicola*	CBS 137324	99.8
	Bt2a/Bt2b	515	MT084117			
	CF1L/CF4	757	MT084121			
PT-5	ITS1/ITS4	525	MT065767	*Penicillium manginii*	CBS 253.31	99.6
	Bt2a/Bt2b	420	MT084118			
PT-6	ITS1/ITS4	502	MT065768	*Aspergillus ustus*	NRRL 275	100
	CF1L/CF4	694	MT084122			
PT-7	ITS1/ITS4	532	MT065769	*Aspergillus tamarii*	NRRL 20818	99.9
	Bt2a/Bt2b	476	MT084119			
	CF1L/CF4	715	MT084123			

^a^GenBank/EMBL/DDBJ accession number

### Evaluation results of theophylline-degrading fungi in solid mediums

The screening was carried out in agar solid mediums for the evaluation of the biocatalytic potential in theophylline degradation. All isolate tea-derived strains were inoculated into an agar solid medium with the presence of dextrose and they were also inoculated into a set of agar solid mediums with increasing theophylline concentrations. The colony diameters of potential theophylline-degrading fungi were measured and showed in Table 3.

**Table 3:** Growth of tea-derived fungi in agar solid medium (2% w/v) with dextrose (2% w/v) (control culture) or presence of theophylline instead of dextrose (30 °C, 5 d, pH 7.0)

Isolate fungi	Colony diameter (cm)			
	Control culture	600 mg/L theophylline	1200 mg/L theophylline	1800 mg/L theophylline
*A. niger*	3.5 ± 0.5	0.5 ± 0.2	No growth	1.0 ± 0.5
*A. sydowii*	2.5 ± 1.0	0.5 ± 0.1	1.0 ± 0.3	0.5 ± 0.3
*A. pallidofulvus*	3.0 ± 0.5	No growth	No growth	0.5 ± 0.1
*A. sesamicola*	3.0 ± 0.5	No growth	No growth	0.5 ± 0.1
*P. mangini*	3.0 ± 1.0	No growth	No growth	No growth
*A. ustus*	2.5 ± 0.5	1.0 ± 0.3	1.5 ± 0.4	1.5 ± 0.4
*A. tamarii*	3.0 ± 0.5	2.0 ± 0.5	2.5 ± 0.5	3.5 ± 1.0

Six isolates could survive in the agar solid mediums (2% w/v) with theophylline. *Aspergillus* spp. showed a better growth in higher evaluated concentrations. Particularly, *A. niger*, *A. sydowii*, *A. ustus* and *A. tamarii* had growth in low theophylline concentration, which showed that these strains had a high utilization ratio of theophylline as carbon source directly [40, 46]. Therefore, *A. niger*, *A. sydowii*, *A. ustus* and *A. tamarii* were considered as potential theophylline-degrading fungi.

### Selection of theophylline-degrading fungi and optimal medium in liquid culture

**Fig. 2:**
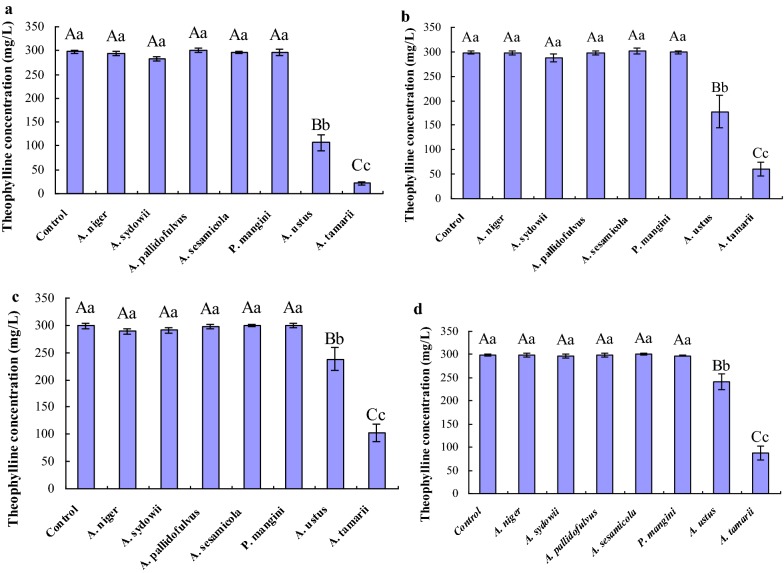
Theophylline degradation capacity of isolate fungi strains in different theophylline liquid mediums. **a***TLM-S* theophylline liquid medium with sucrose as carbon source; **b***TLM-D* theophylline liquid medium with dextrose with sucrose as carbon source; **c***TLM-N* theophylline liquid medium with ammonium sulphate as nitrogen source; **d***TLM-SN* theophylline liquid medium with sucrose and ammonium sulphate as carbon and nitrogen sources, respectively. Biocidal treatment was defined as the control. All data were present by mean value ± SD of three replications. The lowercase letters indicated a significant difference at p < 0.05 levels and the uppercase letters indicated a highly significant difference at p < 0.01 levels using one-way ANOVA of SPSS 20.0. The different letters show significant differences

For theophylline biodegradation in liquid culture, seven isolates were inoculated into TLM with the presence of theophylline and sucrose or dextrose as carbon source, or ammonium sulphate as nitrogen source, respectively. Theophylline concentration and fungal dry mass were determined after cultivation at 30 °C for 5 days. Results are showed in Fig. 2 and Additional file 2: Table S1, respectively. Through comparisons of each isolate, with the presence of carbon source such as sucrose or dextrose, although all isolates could survive and maintain metabolic activity in TLM, theophylline utilization efficiency was different. *A. pallidofulvus*, *A. sesamicola* and *P. mangini* had no ability to use theophylline. Theophylline utilization of *A. niger* and *A. sydowii* was restricted in liquid culture, theophylline removal ratios were about 1.03% and 5.19% in TLM-S, respectively. Only *A. ustus* and *A. tamarii* could utilize caffeine significantly in all given TLM. Hence, *A. ustus*, *A. tamarii*, *A. niger* and *A. sydowii* were potential theophylline-degrading fungi for theophylline degradation in liquid culture.

The presence of additional carbon or nitrogen sources had a significant impact on theophylline degradation and pathway. The optimum liquid medium was chose by comparing theophylline removal ratios in different mediums. In contrast with other mediums (TLM-D, TLM-N and TLM-SN), theophylline degradation level had a highly significant (p < 0.01) improvement in TLM-S. In addition, extra sucrose promoted theophylline degradation in TLM-S inoculated by *A. ustus* and *A. tamarii* through enhancing cell density in liquid culture. Therefore, TLM-S was selected to analyze characterization of theophylline degradation in liquid culture.

### Characterization of theophylline degradation inoculated by theophylline-degrading fungi

**Fig. 3:**
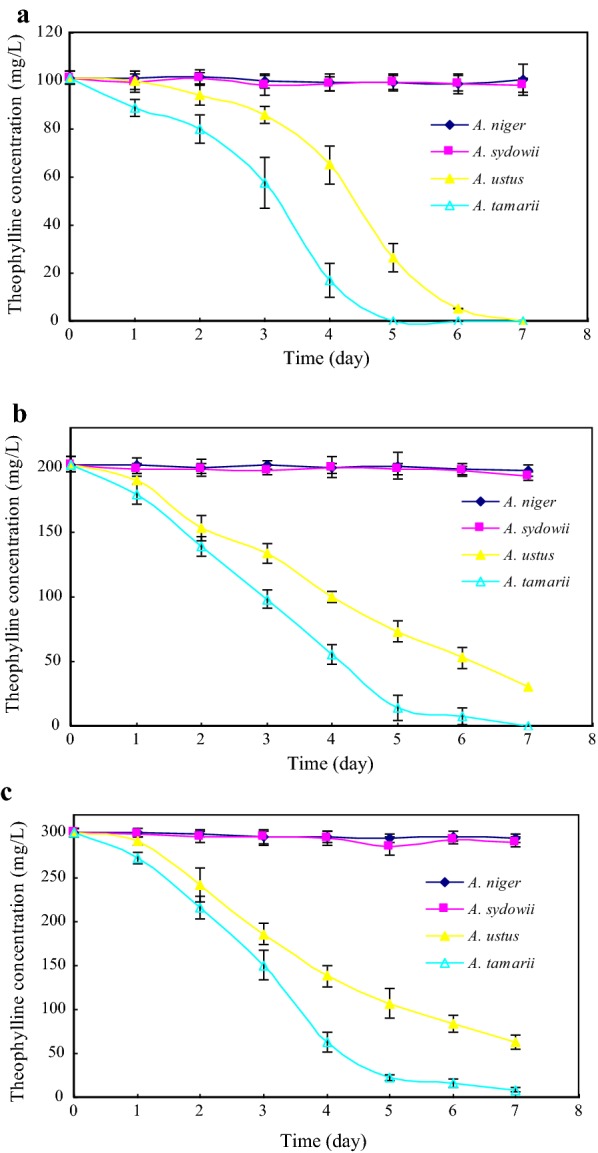
Effects of potential theophylline-degrading fungi on theophylline catabolism in different substrate concentrations. Theophylline concentrations were 100 mg/L (**a**), 200 mg/L (**b**), and 300 mg/L (**c**), respectively. All data were present by mean value ± SD of three replications

*Aspergillus ustus*, *A. tamarii*, *A. niger* and *A. sydowii* were inoculated into TLM-S with increasing theophylline concentrations (100, 200 and 300 mg/L, respectively), and Tissue-culture bottles were incubated in an orbital shaker (130 rpm, 30 °C). The inoculation bottles were took every 24 h for the determination of theophylline and related metabolites by HPLC, and results are presented in Fig. 3. Under effects of *A. ustus* and *A. tamarii*, theophylline decreased highly significantly (p < 0.01) in all substrate concentrations. However, theophylline decreased slightly (p > 0.05) in all concentrations inoculated by *A. niger* and *A. sydowii*. Therefore, *A. ustus* and *A. tamarii* had more advantage in theophylline degradation than *A. niger* and *A. sydowii.* Both *A. ustus and A. tamarii* could degrade theophylline completely in low concentration (100 mg/L theophylline). However, *A. ustus* only degrade 79.00% theophylline in high concentration (300 mg/L theophylline), while *A. tamarii* could degrade theophylline almost completely in all given concentrations, which showed that *A. tamarii* had a higher theophylline degradation capacity.

A series of experiments was conducted to find out theophylline degradation pathway through the identification of catabolic intermediates by HPLC using internal standard method (Table 4). 1,3-Dimethyluric acid, 3-methylxanthine, 3-methyluric acid, xanthine and uric acid were detected consecutively in the liquid culture. 3-Methylxanthine was common and main metabolite through *N*-demethylation at the position N-1 of theophylline in *A. ustus* and *A. tamarii.* Xanthine was a further demethylated metabolite in theophylline degradation found in *A. ustus* and *A. tamarii* through *N*-demethylation at the position N-3 of 3-methylxanthine. In contrast to *A. ustus* that additional metabolites including 1,3-dimethyluric acid and 3-methyluric acid were identified in the culture through the oxidation of theophylline and 3-methylxanthine, respectively, only uric acid was identified in *A. tamarii* culture as the oxidation product of xanthine, which showed the differences in degradation metabolites and pathways between *A. ustus* and *A. tamarii*.

**Table 4:** Theophylline degradation metabolites detected in the liquid culture inoculated by *Aspergillus* fungi

Metabolite	Fungal isolates			
	*A. ustus*	*A. tamarii*	*A. niger*	*A. sydowii*
1,3-Dimethyluric acid	+	−	−	−
1-Methylxanthine	−	−	−	−
3-Methylxanthine	+	+	−	−
1-Methyluric acid	−	−	−	−
3-Methyluric acid	+	−	−	−
Xanthine	+	+	−	−
Uric acid	−	+	−	−

TLM-S inoculated by *Aspergillus* fungi were analyzed by HPLC for 1,3-dimethyluric acid, 1-methylxanthine, 3-methylxanthine, 1-methyluric acid, 3-methyluric acid, xanthine and uric acid

### Production of 3-methylxanthine or xanthine through theophylline degradation

**Fig. 4:**
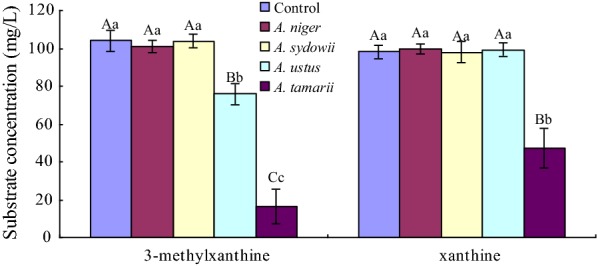
Effects of potential theophylline-degrading fungi on 3-methylxanthine and xanthine, respectively. Biocidal treatment was defined as the control. All data were present by mean value ± SD of three replications. The lowercase letters indicated a significant difference at p < 0.05 levels and the uppercase letters indicated a highly significant difference at p < 0.01 levels using one-way ANOVA of SPSS 20.0. The different letters show significant differences

Several xanthine derivatives including 3-methylxanthine have been synthesized chemically for use in medical industry [47]. Except for engineering a microbial platform for de novo biosynthesis of diverse methylxanthins [48], bioconversion from cheaper feedstocks such as caffeine, theophylline and theobromine was an effective pathway to produce high value methylxanthines via metabolically engineered microorganisms [22]. In this study, 3-methylxanthine and xanthine were common and main products in theophylline degradation by *A. ustus* and *A. tamarii.* Microbial utilization of 3-methylxanthine and xanthine were investigated in liquid culture of isolates. 3-Methylxanthine and xanthine concentrations were determined by HPLC after cultivation for 5 days. As shown in Fig. 4, *A. ustus* and *A. tamarii* had a significant (p < 0.05) or a highly significant (p < 0.01) impact on 3-methylxanthine degradation with a removal ratio of about 27.05% and 84.29%, respectively. Additionally, *A. tamarii* had a highly significant (p < 0.01) impact on xanthine degradation with a removal ratio of about 51.77%. Associated with the metabolites detected in theophylline degradation, 3-methyluric acid and xanthine were 3-methylxanthine degradation metabolites through oxidation and *N*-demethylation, respectively.

**Fig. 5:**
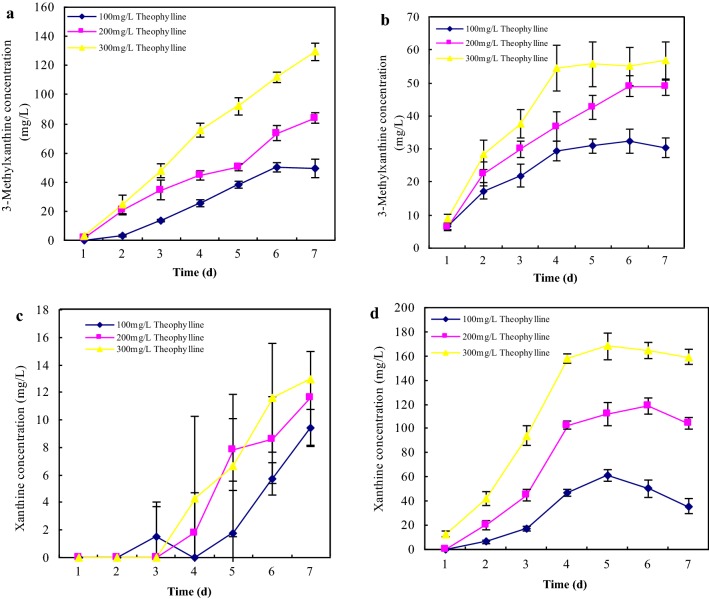
Effects of theophylline concentrations on 3-methylxanthine production (**a**, **b**) and xanthine production (**c**, **d**) by theophylline-degrading fungi. Liquid culture assays were performed using TLM-S with different theophylline concentrations inoculated by *Aspergillus ustus* (**a**, **c**) and *Aspergillus tamarii* (**b**, **d**), respectively. Theophylline concentrations were 100 mg/L (filled rhombus), 200 mg/L (filled square), and 300 mg/L (filled triangle). Concentrations of 3-methylxanthine were present by mean value ± standard deviations (SD) of three replications

Despite of significant impacts on 3-methylxanthine and xanthine degradation, 3-methylxanthine and xanthine concentrations were accumulated largely in TLM-S inoculated by *A. ustus* and *A. tamarii*, respectively. To investigate the application in production of 3-methylxanthine and xanthine by using theophylline-degrading fungi with theophylline as feedstock, quantitative determinations of 3-methylxanthine and xanthine were carried out in all theophylline concentrations inoculated by *A. ustus* and *A. tamarii*, respectively. 3-Methylxanthine and xanthine concentrations in *A. ustus* and *A. tamarii* cultures are presented in Fig. 5. We monitored the accumulation of 3-methylxanthine and xanthine over the course of inoculated culture by *A. ustus* and *A. tamarii*. 3-Methylxanthine was detected in the culture medium after 24 h for the first time, and increased significantly with cultivation. Over a 7-day period cultivation of *A. ustus* (Fig. 5a), 49.68 ± 2.97 mg/L, 83.82 ± 3.35 mg/L and 129.48 ± 5.81 mg/L of 3-methylxanthine were accumulated and increased significantly with increasing initial theophylline concentrations, respectively. Due to high degradation capacity of 3-methylxanthine in *A. tamarii* culture, 3-methylxanthine concentration (Fig. 5b) stayed at a low level that only 56.72 ± 5.81 mg/L of 3-methylxanthine was accumulated in 300 mg/L of theophylline after a 7-day period cultivation. Hence, *A. ustus* exhibited a continuing accumulation of 3-methylxanthine over the course of liquid culture, and increasing initial theophylline concentrations could improve the production of 3-methylxanthine.

Xanthine concentration over a 7-day period cultivation of *A. ustus* (Fig. 5c) maintained at a low level below 15.00 mg/L in all substrate concentrations. However, Fig. 5d showed a reaction containing theophylline in *A. tamarii* culture provided linear conversion of theophylline to xanthine. Over a 7-day period cultivation of *A. tamarii*, 35.88 ± 6.65 mg/L, 103.95 ± 4.82 mg/L and 159.11 ± 10.8 mg/L of xanthine were accumulated and increased significantly with increasing initial theophylline concentrations through *N*-demethylation at the position N-3 of 3-methylxanthine, respectively. Therefore, xanthine was main metabolite in theophylline degradation over the course of *A. tamarii* liquid culture, which showed that *A. tamarii* could be used for the production of xanthine with theophylline as feedstock through microbial conversion.

## Discussion

Although caffeine and related methylxanthines are toxic to most bacteria and invertebrates [49], some bacteria and fungi have evolved the ability to metabolize caffeine [12, 16]. In our previous studies [40–42], caffeine content was decreased significantly, and *A. sydowii* leaded to caffeine degradation and converted the most degraded caffeine to theophylline. In this study, we confirmed a new phenomenon (Fig. 1) that theophylline degradation was found in SSF of pu-erh tea. This suggested that potential theophylline-degrading fungi could be found in fungal community.

Molecular identification of fungi is mostly dependent on PCR amplified sequences of ITS, β-tubulin and calmodulin genes, particularly the genera *Aspergillus* and *Penicillium* [50, 51]. The amplified sequencing and colony morphology indicated that their seven candidate isolates were identified specifically as *A. niger*, *A. sydowii*, *A. pallidofulvus*, *A. ustus*, *A. sesamicola*, *A. tamarii* and *P. mangini*, respectively. This was in line with the observation in literature that *Aspergillus* spp. and *Penicillium* spp. are the filamentous fungal genera commonly associated with SSF of pu-erh tea [52–54], particularly *A. niger*, *A. sydowii* and *A. tamarii* having been widely reported as the dominant fungi in the SSF of pu-erh tea.

The assessment in agar mediums with theophylline as carbon source showed that six *Aspergillus* isolates could survive in theophylline agar mediums, which suggested that those six candidate isolates (Table 3) could utilize theophylline as potential carbon source directly in the absence of other carbon source. The data from the liquid culture inoculated by seven isolates indicated that *A. ustus* and *A. tamarii* could degrade theophylline significantly (p < 0.05) or highly significantly (p < 0.01); moreover, the theophylline utilization of *A. niger* and *A. sydowii* were restricted. However, at the presence of sucrose as carbon source in liquid culture, *A. pallidofulvus, A. sesamicola* and *P. mangini* had no ability to use theophylline. Though the effect of C/N ratio on growth is strain-dependent [55], increasing C/N ratio would generally favour fungal growth, which influenced microbial metabolism to a certain degree. Through comparisons of theophylline degradation capacity in different TLM containing sucrose or dextrose as carbon source, or ammonium sulphate as nitrogen source, sucrose enhanced theophylline degradation highly significantly (p < 0.01) through improving fungal growth in liquid culture. Therefore, TLM-S medium was the optimization for theophylline degradation to analyze theophylline degradation metabolites in liquid culture.

**Fig. 6:**
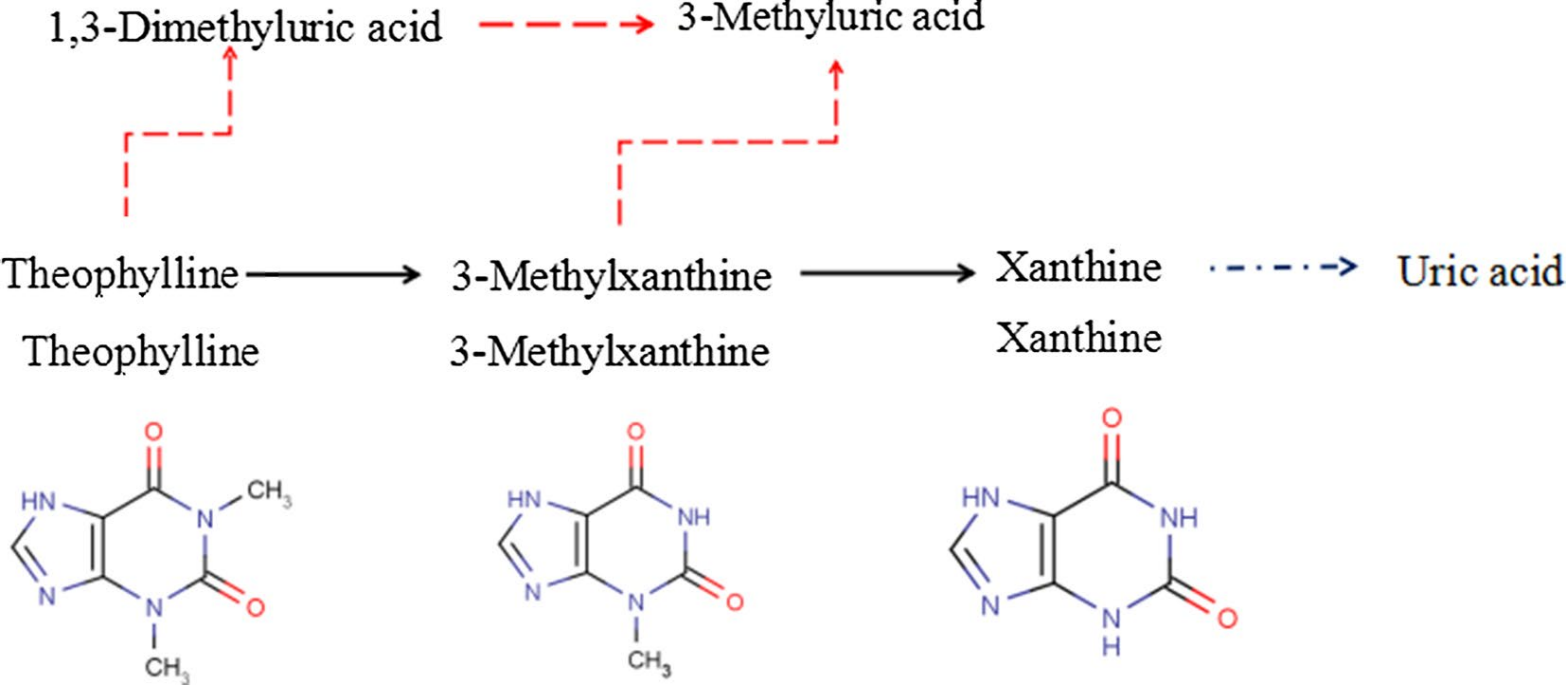
Hypothetical theophylline degradation pathways employed by *A. ustus* and *A. tamarii*. The black arrows indicate the common pathways to *A. ustus* and *A. tamarii*. The red arrows indicate a pathway detected for *A. ustus* only. The blue arrows indicate a pathway detected for *A. tamarii* only

Except for caffeine, theophylline and theobromine are main purine alkaloid in tea, which both have close connection with caffeine metabolism that theophylline is catabolite of caffeine and theobromine is precursor of caffeine biosynthesis in *Camellia sinensis*. As mentioned above, the pathway of caffeine degradation metabolism is comparatively clear in microorganisms that both *N*-demethylation and oxidation were found in caffeine degradation, and *N*-demethylation was main pathway in fungi [17, 18, 56]. In addition, several bacteria and fungi have been shown to be able to utilize or degrade theobromine, which include *P. putida*, *A. niger*, *Talaromyces marneffei*, and *Talaromyces verruculosus* [12, 18, 20]. Mensah et al. confirmed the existence of subsequent demethylation and oxidation in theobromine degradation through the detection of correlative metabolites by HPLC under the effects of theobromine-degrading fungi [20]. However, theophylline degradation pathways and metabolites were not completely clear in fungi. In this study, it is confirmed that *A. ustus* and *A. tamarii* isolated from pu-erh tea could degrade theophylline in liquid culture. Based on the downstream metabolites detected in liquid culture, hypothetical theophylline degradation pathways were established and shown in Fig. 6. Both *N*-demethylation and oxidation were theophylline degradation pathways found in *A. ustus* and *A. tamarii* culture. Theophylline and related demethylated metabolites can be oxidized to 1,3-dimethyluric acid, 3-methyluric acid and uric acid, respectively. The degradation metabolites suggested the differences in degradation pathways of *A. ustus* and *A. tamari*, except the common pathway that theophylline → 3-methylxanthine → xanthine. Therefore, *N*-demethylation was main theophylline degradation pathway, which was similar to caffeine and theobromine catabolism in fungi.

3-Methylxanthine and xanthine were common downstream metabolites detected in *A. ustus* and *A. tamarii* cultures. 3-Methylxanthine and other xanthine derivatives have been shown various biomedical effects as adenosine antagonist and inhibitors of Primary Amine Oxidase [57, 58]. Besides chemical synthesis, biotransformation and biosynthesis offered alternative way to produce 3-methylxanthine and other xanthine derivatives [22, 48]. Due to theophylline degradation characteristic, *A. ustus* and *A. tamarii* would be applied in the production of 3-methylxanthine and xanthine with theophylline as feedstock through microbial conversion. The differences in accumulated concentrations of 3-methylxanthine and xanthine suggested that *A. ustus* benefits the production of 3-methylxanthine, while *A. tamarii* benefits the production of xanthine. After a 7-day period cultivation in 300 mg/L of TLM, *A. ustus* could produce 129.48 ± 5.81 mg/L of 3-methylxanthine, and *A. tamarii* could produce 159.11 ± 10.8 mg/L of xanthine, respectively.

## Conclusions

This paper describes theophylline degradation pathways in tea-derived fungi and explores the application in production of methylxanthines. *A. ustus* and *A. tamarii* isolated from SSF of pu-erh tea and identified based on ITS, β-tubulin and calmodulin gene sequences, were confirmed to degrade theophylline significantly (p < 0.01) in liquid culture through the sequential selections. Extensive experiments were carried out to detect related degradation metabolites by using HPLC, finding *N*-demethylation and oxidation in theophylline catabolism. Through the absolute quantitative detection, it is showed that 3-methylxanthine and xanthine were main degraded metabolites in *A. ustus* and *A. tamarii* respectively, which suggests that *A. ustus* benefits the production of 3-methylxanthine, while *A. tamarii* benefits the production of xanthine with theophylline as feedstock. This paper also suggests theophylline degradation pathway in *Aspergillus* fungi and represents a new microbial synthesis platform for production of methylxanthines using theophylline through the inoculation of *A. ustus* and *A. tamarii*, respectively.

## Supplementary information

**Additional file 1: Figure S1.** Colony characteristics (a, b) and conidial structure (c, d) of strain PT-6. (a): Front on PDA medium at 25 °C for 7 days. (b): Back on PDA medium at 25 °C for 7 days. (c): Mature conidia heads, conidia stems and antipodal cells (200 ×). (d): Hyohae, conidia stems and conidiums (400 ×). **Figure S2.** Colony characteristics (a, b) and conidial structure (c, d) of strain PT-7. (a): Front on PDA medium at 25 °C for 7 days. (b): Back on PDA medium at 25 °C for 7 days. (c): Conidia heads(200 ×). (d): Conidia stems, sterigmas and conidiums (400 ×). **Figure S3.** The received sequences of strain PT-6 (502 bp ITS sequence and 694 bp calmodulin sequence). **Figure S4.** The received sequences of strain PT-7 (532 bp ITS sequence,476 bp β-tubulin sequence and 715 bp calmodulin sequence). **Figure S5.** Neighbor-Joining consensus trees of (a) *Aspergillus ustus* PT-6 and (b) *Aspergillus tamarii* PT-7. Identification was based on ITS and calmodulin genes for *A. ustus* PT-6, and ITS, β-tubulin and calmodulin genes for *A. tamarii* PT-7. The numbers over branches represent bootstrap confidence values (%) based on 1000 replicates. The scale bar denotes the nucleotide substitution per sequence.

**Additional file 2: Table S1.** Comparisons of fungal dry mass (mg) of each isolate in different theophylline liquid mediums after cultivation at 30 °C for 5 days. TLM-S = theophylline liquid medium with sucrose as carbon source; TLM-D = theophylline liquid medium with dextrose with sucrose as carbon source; TLM-N = theophylline liquid medium with ammonium sulphate as nitrogen source; TLM-SN = theophylline liquid medium with sucrose and ammonium sulphate as carbon and nitrogen sources, respectively. All data were present by mean value ± SD of three replications. The lowercase letters indicated a significant difference at p < 0.05 levels and the uppercase letters indicated a highly significant difference at p < 0.01 levels by using one-way ANOVA of SPSS 20.0. The different letters show significant differences of each isolate between different theophylline liquid mediums.

## Data Availability

The data that support the findings of this study are available from the corresponding author upon reasonable request.

## Abbreviations

SSF: Solid-state fermentation
CFU: Colony forming units
HPLC: High-performance liquid chromatography
PDA: Potato dextrose agar
PCR: Polymerase chain reaction
ITS: Internal transcribed spacer
dNTPs: Deoxy-ribonucleoside triphosphates
TLM: Theophylline liquid medium
TLM-S: Theophylline liquid medium with sucrose as carbon source
TLM-D: Theophylline liquid medium with dextrose as carbon source
TLM-N: Theophylline liquid medium with ammonium sulphate as nitrogen source
TLM-SN: Theophylline liquid medium with sucrose and ammonium sulphate as carbon and nitrogen sources
SPSS: Statistical product and service solutions
SD: Standard deviation
ANOVA: Analysis of variance
