# Supplementary material for: Isolation, characterization and application of theophylline-degrading Aspergillus fungi
Source: Microb Cell Fact. 2020 Mar 19;19:72. doi: 10.1186/s12934-020-01333-0 (PMC7082937; doi:10.1186/s12934-020-01333-0)
Supplement: Supplementary file 1 — Additional file 1: Figure S1. Colony characteristics (a, b) and conidial structure (c, d) of strain PT-6. (a): Front on PDA medium at 25 °C for 7 days. (b): Back on PDA medium at 25 °C for 7 days. (c): Mature conidia heads, conidia stems and antipodal cells (200 ×). (d): Hyohae, conidia stems and conidiums (400 ×). Figure S2. Colony characteristics (a, b) and conidial structure (c, d) of strain PT-7. (a): Front on PDA medium at 25 °C for 7 days. (b): Back on PDA medium at 25 °C for 7 days. (c): Conidia heads(200 ×). (d): Conidia stems, sterigmas and conidiums (400 ×). Figure S3. The received sequences of strain PT-6 (502 bp ITS sequence and 694 bp calmodulin sequence). Figure S4. The received sequences of strain PT-7 (532 bp ITS sequence,476 bp β-tubulin sequence and 715 bp calmodulin sequence). Figure S5. Neighbor-Joining consensus trees of (a) Aspergillus ustus PT-6 and (b) Aspergillus tamarii PT-7. Identification was based on ITS and calmodulin genes for A. ustus PT-6, and ITS, β-tubulin and calmodulin genes for A. tamarii PT-7. The numbers over branches represent bootstrap confidence values (%) based on 1000 replicates. The scale bar denotes the nucleotide substitution per sequence. [file 12934_2020_1333_MOESM1_ESM.pdf]

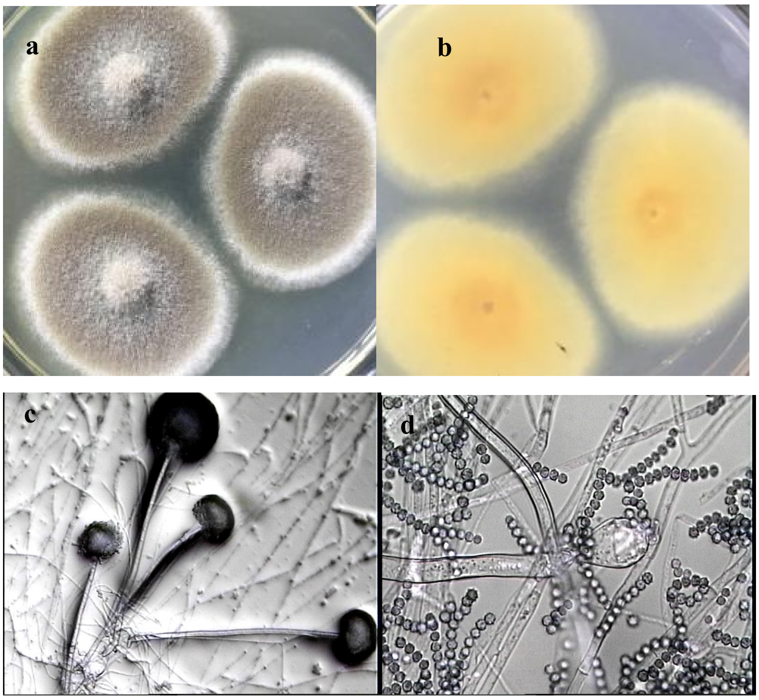

Additional file 1: Figure S1. Colony characteristics (a, b) and conidial structure (c, d) of strain PT-6.

(a): Front on PDA medium at 25 ℃ for 7 days. (b): Back on PDA medium at 25 ℃ for 7 days.

(c): Mature conidia heads, conidia stems and antipodal cells (200×). (d): Hyohae, conidia stems and conidiums (400×).

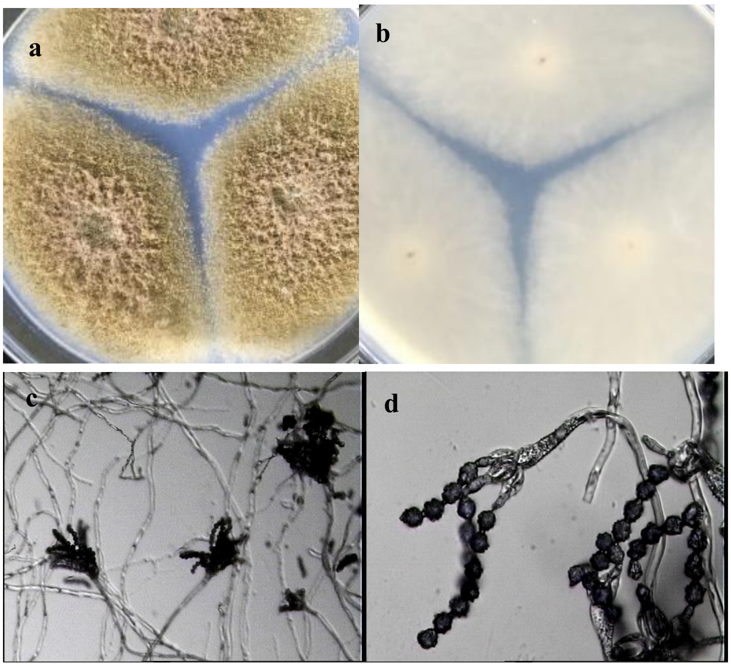

Additional file 1: Figure S2. Colony characteristics (a, b) and conidial structure (c, d) of strain PT-7.

(a): Front on PDA medium at 25 ℃ for 7 days. (b): Back on PDA medium at 25 ℃ for 7 days.

(c) Conidia heads(200×). (d): Conidia stems, sterigmas and conidiums (400×).

ITS sequence of strain PT-6 (502 bp):
1ACCCGTGAATACCTGACCAACGTTGCTTCGGCGGTGCGCTCCCCCCGGGG50
51GCAGCCGCCGGAGACCACACCGAACCTCTTGTTATAGCGTGTCGTCTGAG100
101CTTGATACAAGCAAACCTAATTAAAACTTTCAACAATGGATCTCTTGGTT150
151CCGGCATCGATGAAGAACGCAGCGAACTGCGATAAGTAATGTGAATTGCA200
201GAATTCAGTGAATCATCGAGTCTTTGAACGCACATTGCGCCCCCTGGCAT250
251TCCGGGGGGCATGCCTGTCCGAGCGTCATTGCTGCCCTTCAAGCCCGGCT300
301TGTGTGTTGGGTCGTCGTCCCCCCCGGGGGACGGGCCCGAAAGGCAGCGG350
351CGGCACCGCGTCCTGGTCCTCGAGCGTATGGGGCTTTGTCACCCGCTCGT400
401TTAGGGCCGGCCGGGCGCCAGCCGGCGTCTCCAAACCTTTTATTTTACCA450
451GGTTGACCTCGGATCAGGTAGGGATACCCGCTGAACTTAAGCATATCAAT500
501AA502
Calmodulin sequence of strain PT-6 (694 bp):
1TTTGTAGTCCCATATGGTATTACATAAACTACCGGTCGCCATTTAAGAAT50
51CACTAACTTACCCTTTTCCGCGTGAAACAGGACAAGGATGGCGATGGTTA100
101GTGCAATTCTCGCGCCCATTTATTCCGGCGATGTTATCGCATACGCCCTA150
151AAAATATCCAGGAAATTTGGTCGCATTTACTGCTCTGGTATTAATATATG200
201CGTGTAGGCCAGATCACCACCAAGGAACTCGGCACTGTGATGCGCTCACT250
251CGGCCAGAATCCCTCCGAGTCCGAACTTCAGGACATGATCAACGAGGTCG300
301ACGCCGACAACAATGGCACCATTGACTTTCCAGGTATTCTATTCGGCTGT350
351TTTTTTCGTGTGCGATACAATTGGGCAGATCACTGATATTCAAACCAGAG400
401TTCCTCACAATGATGGCCAGAAAGATGAAGGACACCGACTCCGAGGAGGA450
451AATCCGCGAGGCGTTCAAGGTTTTCGACCGTGACAATAATGGTTTCATCT500
501CGGCTGCCGAGCTACGCCATGTCATGACCTCTATTGGCGAGAAGCTCACC550
551GATGATGAAGTCGATGAGATGATCCGTGAGGCAGACCAGGATGGTGACGG600
601CCGAATTGACTGTACGTTGACCGTTCCAGTTATACAGTTATACTCGATGT650
651GCAAGAATTGATGCTAATAATTCTTCAGACAACGAATTCGTCCA694

Additional file 1: Figure S3. The received sequences of strain PT-6 (502 bp ITS sequence and 694 bp calmodulin sequence).

ITS sequence of strain PT-7 (532 bp):
1CCTCCCACCCGTGTTTACTGTAACCTTAGTTGCTTCGGCGGGCCCGCCTT50
51TAAGGCCGCCGGGGGGCATCAGCCCCCGGGCCCGCGCCCGCCGGAGACAC100
101CACGAACTCTGTCTGATCTAGTGAAGTCTGAGTTGATTGTATCGCAATCA150
151GTTAAAACTTTCAACAATGGATCTCTTGGTTCCGGCATCGATGAAGAACG200
201CAGCGAAATGCGATAACTAGTGTGAATTGCAGAATTCCGTGAATCATCGA250
251GTCTTTGAACGCACATTGCGCCCCCTGGTATTCCGGGGGGCATGCCTGTC300
301CGAGCGTCATTGCTGCCCATCAAGCACGGCTTGTGTGTTGGGTCGTCGTC350
351CCCTCTTCGGGGGGGACGGGCCCCAAAGGCAGCGGCGGCACCGCGTCCGA400
401TCCTCGAGCGTATGGGGCTTTGTCACCCGCTCTGTAGGCCCGGCCGGCGC450
451TTGCCGAACGCAAAACAACCATTCTTTCCAGGTTGACCTCGGATCAGGTA500
501GGGATACCCGCTGAACTTAAGCATATCAATAA532
β-tubulin sequence of strain PT-7 (476 bp):
1CAAGGAACTGCACAGAAGCATGAACTCAGATGTGCCCTACTGTGTCTGCC50
51ACGTGTTTGCTAACATCTTTGCAGGCAGACCATCTCTGGCGAGCACGGCC100
101TTGACGGCTCCGGTGTGTAAGTACAATCCGTGTACACCTCGAACGAACGA150
151CAACCAGATGGCATTGGAAGAGTTGGAATGGGTCTGACGGGAAGGATAGT200
201TACAATGGCTCCTCCGATCTCCAGCTGGAGCGTATGAACGTCTACTTCAA250
251CGAGGTGCGTACCTCACATTTTTCAGCCTCTTTGACAACGCTTTGCAAGT300
301CCTGACCGCTTCTCCAGGCCAGCGGAAACAAGTATGTCCCTCGTGCCGTC350
351CTTGTCGATCTTGAGCCCGGTACCATGGACGCCGTCCGTGCCGGTCCCTT400
401CGGTCAGCTGTTCCGTCCCGACAACTTCGTTTTCGGCCAGTCCGGTGCTG450
451GTAACAACTGGGCCAAGGGTCACTAC476
Calmodulin sequence of strain PT-7 (715 bp):
1GTCATGAACGTCGTTCGCGAAAATCGGCTTTGTGAGTAGACTTTATTTGA50
51ACACAAGCTGACTGGGCTTCTCTTGGGTTTCCTATAGGACAAGGACGGTG100
101ATGGTTAGTACAGTCTCTTTCATTCCGTCTCCCTTCAAATGCGACCAGTA150
151TCTTTTAGCCGGCATAGTTTTATCCATTTTCTGTTCGATCGGCTGAAGTC200
201TTTGGCATTGATGGATTGACTTGATATGCAGGCCAGATCACCACCAAGGA250
251GTTGGGCACTGTCATGCGCTCTCTGGGCCAAAACCCCTCTGAGTCGGAAC300
301TCCAGGACATGATTAACGAAGTTGACGCCGATAACAATGGCACCATTGAC350
351TTTCCTGGTACGAGAGGGCTTCCGTACATTTTACAAATAAAATAGCTGTT400
401AATGTTCAACCAGAGTTCCTCACGATGATGGCGAGAAAGATGAAGGATAC450
451CGACTCTGAGGAGGAGATCCGGGAGGCTTTCAAGGTTTTCGACCGCGATA500
501ACAACGGTTTCATCTCCGCTGCCGAATTGCGCCACGTCATGACCTCCATC550
551GGCGAGAAGCTTACCGATGACGAAGTTGATGAGATGATCCGCGAGGCGGA600
601TCAGGATGGTGACGGTCGGATCGACTGTATGTTTCGAGAAGCCCTCCCAC650
651ACACACCTATTGCGGCTGTGAAACCGGTGATACTGATCGATTTAGACAA700
701CGAGTTCGTCCAACT715

Additional file 1: Figure S4. The received sequences of strain PT-7 (532 bp ITS sequence,476 bp β-tubulin sequence and 715 bp calmodulin sequence).

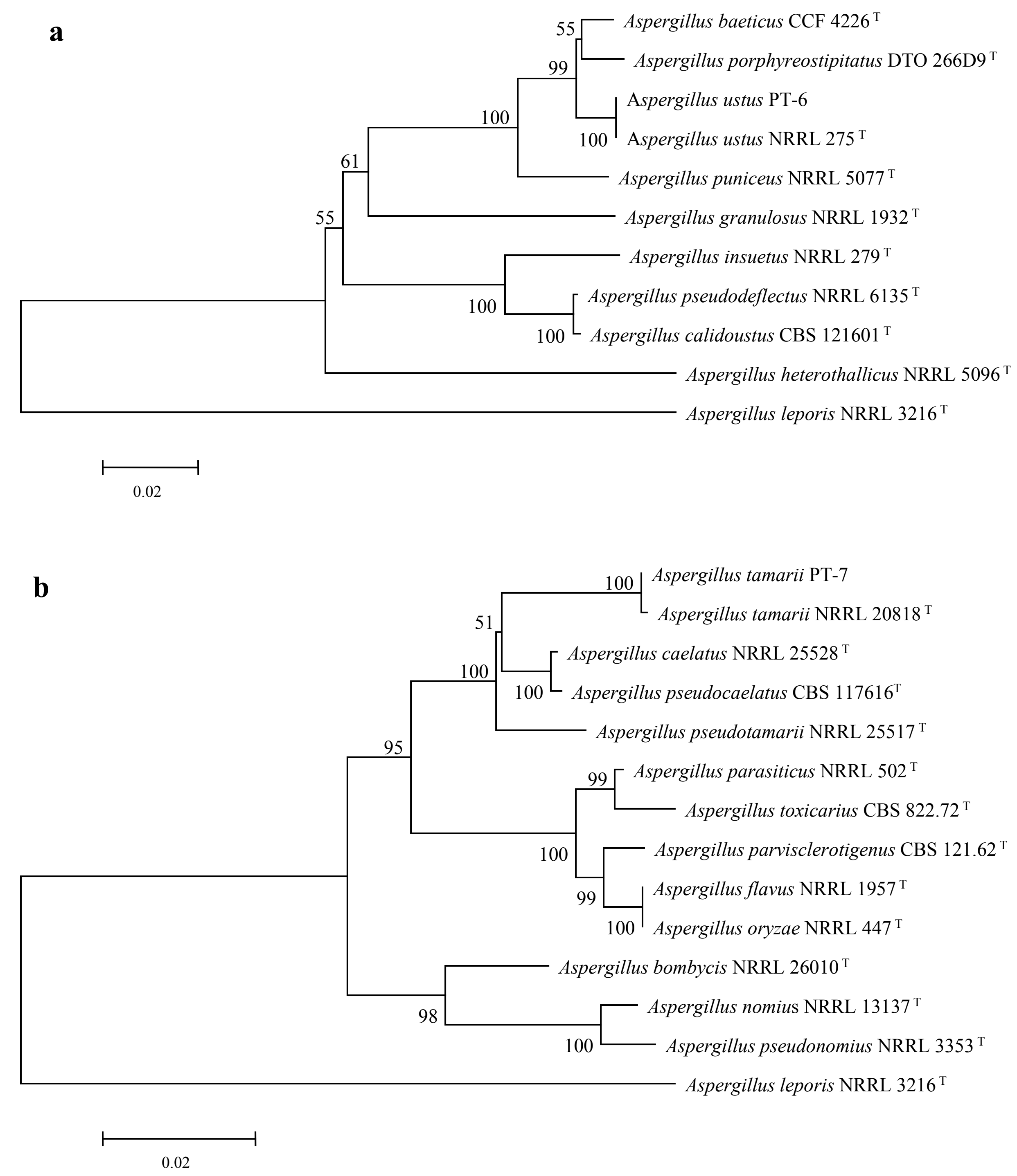

Additional file 1: Figure S5. Neighbor-Joining consensus trees of (a) *Aspergillus ustus* PT-6 and (b) *Aspergillus tamarii* PT-7.

Identification was based on ITS and calmodulin genes for *A. ustus* PT-6, and ITS, β-tubulin and calmodulin genes for *A. tamarii* PT-7. The numbers over branches represent bootstrap confidence values (%) based on 1000 replicates. The scale bar denotes the nucleotide substitution per sequence.
