# Supplementary material for: Isolation, characterization and application of theophylline-degrading Aspergillus fungi
Source: Microb Cell Fact. 2020 Mar 19;19:72. doi: 10.1186/s12934-020-01333-0 (PMC7082937; doi:10.1186/s12934-020-01333-0)
Supplement: Supplementary file 2 — Additional file 2: Table S1. Comparisons of fungal dry mass (mg) of each isolate in different theophylline liquid mediums after cultivation at 30 °C for 5 days. TLM-S = theophylline liquid medium with sucrose as carbon source; TLM-D = theophylline liquid medium with dextrose with sucrose as carbon source; TLM-N = theophylline liquid medium with ammonium sulphate as nitrogen source; TLM-SN = theophylline liquid medium with sucrose and ammonium sulphate as carbon and nitrogen sources, respectively. All data were present by mean value ± SD of three replications. The lowercase letters indicated a significant difference at p < 0.05 levels and the uppercase letters indicated a highly significant difference at p < 0.01 levels by using one-way ANOVA of SPSS 20.0. The different letters show significant differences of each isolate between different theophylline liquid mediums. [file 12934_2020_1333_MOESM2_ESM.doc]

**Additional file 2: Table S1. Comparisons of fungal dry mass (mg) of each isolate in different theophylline liquid mediums after cultivation at 30 °C for 5 days.**

| Factor | TLM-S | TLM-D | TLM-N | TLM-SN |
| --- | --- | --- | --- | --- |
| *A. niger* | 264.0 ± 33.9^Aa^ | 228.0 ± 11.9 ^Aa^ | 226.8 ± 9.6^Aa^ | 253.8 ± 41.5^Aa^ |
| *A. sydowii* | 202.7±19.9^Aa^ | 184.7 ± 7.6^ABa^ | 186.2 ± 17.0^ABa^ | 145.5 ± 11.0^Bb^ |
| *A. ustus* | 297.7 ± 13.1^Aa^ | 275.7 ± 11.8^Abc^ | 267.1 ± 11.0^Ac^ | 294.3 ± 7.3^Aab^ |
| *A. tamarii* | 314.5 ± 19.9^Aa^ | 299.7 ± 5.2^Aab^ | 287.0 ± 7.1^Ab^ | 297.0 ± 9.4^Aab^ |
| *A. pallidofulvus* | 222.8 ± 14.2^Aa^ | 220.0 ± 25.0^Aa^ | 203.0 ± 12.6^Aa^ | 210.0 ± 13.1^Aa^ |
| *A. sesamicola* | 232.8 ± 16.8^Aa^ | 208.0 ± 15.3^Aab^ | 183.7 ± 22.9^Ab^ | 208.3 ± 14.5^Aab^ |
| *P. mangini* | 185.5 ± 22.7^Aa^ | 184.0 ± 32.8^Aa^ | 151.3 ± 23.9^Aa^ | 200.7 ± 15.0^Aa^ |

TLM-S = theophylline liquid medium with sucrose as carbon source; TLM-D = theophylline liquid medium with dextrose with sucrose as carbon source; TLM-N = theophylline liquid medium with ammonium sulphate as nitrogen source; TLM-SN = theophylline liquid medium with sucrose and ammonium sulphate as carbon and nitrogen sources, respectively. All data were present by mean value ± SD of three replications.

The lowercase letters indicated a significant difference at p < 0.05 levels and the uppercase letters indicated a highly significant difference at p < 0.01 levels by using one-way ANOVA of SPSS 20.0. The different letters show significant differences of each isolate between different theophylline liquid mediums .
